# YOLO–LaserGalvo: A Vision–Laser-Ranging System for High-Precision Welding Torch Localization

**DOI:** 10.3390/s25206279

**Published:** 2025-10-10

**Authors:** Jiajun Li, Tianlun Wang, Wei Wei

**Affiliations:** 1 Faculty of Applied Sciences, Macao Polytechnic University, Macao 999078, China; 2 School of Automation Engineering, University of Electronic Science and Technology of China, Chengdu 611731, China; 2019060508020@std.uestc.edu.cn

**Keywords:** welding torch localization, vision-based positioning, laser galvanometer scanning, deep learning detection, engineering education

## Abstract

A novel closed loop visual positioning system, termed YOLO–LaserGalvo (YLGS), is proposed for precise localization of welding torch tips in industrial welding automation. The proposed system integrates a monocular camera, an infrared laser distance sensor with a galvanometer scanner, and a customized deep learning detector based on an improved YOLOv11 model. In operation, the vision subsystem first detects the approximate image location of the torch tip using the YOLOv11-based model. Guided by this detection, the galvanometer steers the IR laser beam to that point and measures the distance to the torch tip. The distance feedback is then fused with the vision coordinates to compute the precise 3D position of the torch tip in real-time. Under complex illumination, the proposed YLGS system exhibits superior robustness compared with color-marker and ArUco baselines. Experimental evaluation shows that the system outperforms traditional color-marker and ArUco-based methods in terms of accuracy, robustness, and processing speed. This marker-free method provides high-precision torch positioning without requiring structured lighting or artificial markers. Its pedagogical implications in engineering education are also discussed. Potential future work includes extending the method to full 6-DOF pose estimation and integrating additional sensors for enhanced performance.

## 1. Introduction

Vision-guided robotic welding has become indispensable in modern industrial automation, where the precise localization of the welding torch directly determines seam quality and process efficiency [1]. Accurate positioning of the torch tip underpins seam tracking and collision avoidance in automatic welding systems. Traditional pose estimation approaches typically rely on external fiducials or simple edge cues. For instance, ArUco markers and colored tapes affixed to the torch handle or nozzle are often used as reference points during welding [2,3]. However, these methods require careful placement and calibration and are prone to occlusion by spatter, workpiece motion, or mechanical vibration [4]. Likewise, color- or edge-based detectors depend on strong visual contrast and can fail under illumination changes and intense arc glare.

Recent advances in computer vision and deep learning have opened new avenues for robust target detection in complex industrial scenes. One-stage object detectors—such as convolutional neural network-based YOLO models—have achieved impressive real-time detection and localization performance in cluttered backgrounds [5,6]. Compared with marker-based techniques, these data-driven visual methods offer greater adaptability and generality, learning to recognize the torch tip and its surroundings across diverse conditions. Nevertheless, purely visual approaches still face challenges: arc glare, specular reflections from metal surfaces, and environmental clutter can degrade detection; moreover, monocular vision provides only two-dimensional evidence and lacks direct depth, and this depth ambiguity can introduce significant errors in three-dimensional position estimation [7,8].

To overcome limitations in robustness, real-time performance, and accuracy, this study develops—for the first time—a YOLO–LaserGalvo (YLGS) system that realizes vision-driven closed loop control. A monocular camera with an improved YOLOv11 detector provides pixel-level hypotheses of the torch tip location; a high-bandwidth galvanometer then steers an infrared ranging beam to the predicted pixel, and the measured range is fused with image geometry to recover metric 3D tip coordinates. A small ArUco tag is used only to bootstrap the initial pose and is not required during normal operation, mitigating the fragility of fully marker-based schemes. By closing the perception–actuation loop and compensating galvo angles with real-time distance, YLGS achieves millimeter-level localization under arc glare and clutter while maintaining real-time throughput, offering a flexible, sensor-lean alternative to contact probes.

## 2. Related Work

### 2.1. Overview

In welding automation, fixture imperfections and thermally induced deformation often cause the torch end-effector to deviate from its programmed/nominal pose, motivating the use of vision sensors for real-time pose calibration and localization. Existing studies predominantly leverage diverse vision-based strategies to measure the torch tip or construct reference tools to obtain high-precision six-degree-of-freedom (6-DOF) poses. Le et al. [3] developed the Solpen locator, which attaches ArUco codes to a 3D-printed 31-faced polyhedron; with a single standard camera, the tool’s 6D pose is stably estimated via the PnP algorithm, achieving millimeter-level accuracy. Garrido-Jurado et al. [9] proposed a robust ArUco marker system capable of reliable detection even under partial occlusion. RGB–D–based weld-seam extraction has been widely explored for robotic welding automation. Gómez-Espinosa et al. [2] pre-placed colored patches on the workpiece, acquired RGB–D point clouds using a RealSense D435 camera, and fused depth with HSV segmentation to rapidly recover the 3D weld trajectory; similarly, Kim et al. [10] extracted multiple weld seams directly from RGB–D images to support robotic arc-welding tasks, demonstrating the effectiveness of depth-assisted fusion for weld localization and tracking.

Active-vision approaches are likewise widely adopted for welding localization, offering high-accuracy 3D profile measurements and serving as auxiliary illumination to extract weld or workpiece features [11]. The literature indicates that active perception typically employs structured light and tunes illumination intensity and wavelength to enhance robustness [12,13,14]. For example, Zhou et al. [15] mounted a SmartRay ECCO95.040 laser sensor at the torch end, generated weld point clouds via high-resolution scanning, and improved localization through multi-frame clustering; together with Zhang’s calibration, PnP, and hand–eye calibration plus triangulation, the 3D pose of the torch end was updated in real-time.

In recent years, depth camera fusion methods have gained attention [16]. By projecting infrared structured light, depth cameras superimpose distance information onto RGB imagery, enabling direct depth measurement of spatial points [17]. In welding applications, such cameras not only identify welds or fiducials but also help estimate the torch–workpiece standoff distance [18]. For instance, Li et al. [19] propose an improved-YOLOv5 guidance system that uses a RealSense D435i to detect the weld center; by combining the bounding-box center pixel with depth, the true 3D position in the camera frame is computed. The robot is first guided toward the weld, after which a laser vision sensor finely tracks the torch along the seam. This hybrid scheme couples the coarse, wide-FOV localization of a depth camera with the high accuracy of a laser sensor, ultimately enabling human-free welding operations.

### 2.2. Limitations of Existing Work

Despite notable progress, current systems often fall short of the reliability and accuracy required on industrial shop floors. First, robustness is a pervasive issue: arc light, slag, and fumes perturb sensors and amplify feature-extraction errors, forcing many systems to rely on additional shielding or human intervention for stable operation [20,21]. Second, range-accuracy bottlenecks arise in triangulation-based setups, depth error grows rapidly with distance—structured light cameras degrade sharply at meter-scale ranges—whereas permissible weld tolerances are typically only a few millimeters [22]. Furthermore, many systems lack closed loop control; sensing and control are loosely coupled, so trajectory deviations due to workpiece or sensor drift are not corrected in time. Commercial welding-vision products still often require manual intervention to complete an entire seam, and much of the research focuses on isolated modules rather than unified perception–decision–actuation architectures [23,24,25]. As Li et al. [19] note, even when a laser sensor tracks the weld, robot approach to the workpiece still needs manual calibration, evidencing a break in the sensing–control chain. In sum, truly automatic torch localization demands improved harsh-environment resilience, long-range ranging accuracy, and a tightly integrated closed loop perception-control system.

## 3. Methods

### 3.1. System Overview

**Figure 1:**
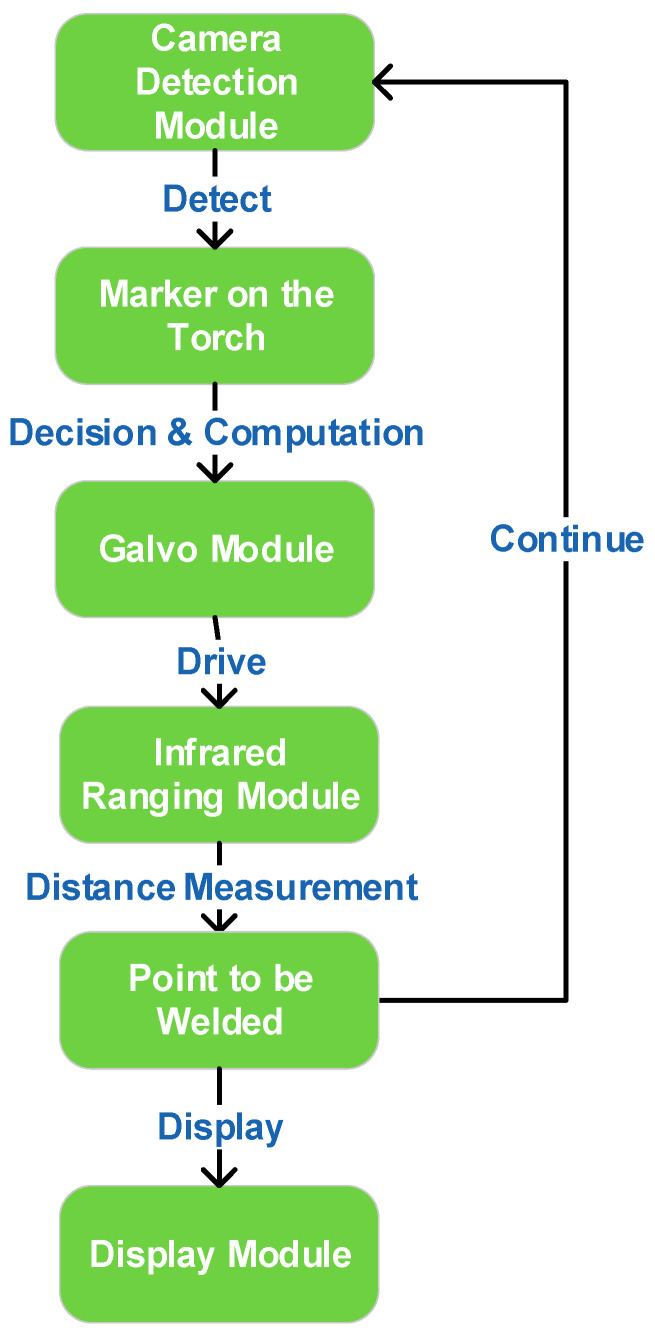
System workflow.

The system comprises four core modules: camera module, galvo module, laser-ranging module, and display module. As illustrated in Figure 1, the workflow proceeds as follows: the camera module acquires frames and cooperates with the improved YOLO detector to identify the torch-mounted markers and the IR spot in pixel coordinates; the controller then computes the required galvo X/Y deflections to steer the beam from the current location A to the desired pixel B; once the spot is stably placed on the target pixel, the laser-ranging module returns the metric distance at that point; finally, the display module visualizes the localization result in the UI and highlights the corresponding hole index and status. This establishes a perception–decision–actuation closed loop that links detection, ranging, and control to the overall objective of real-time, millimeter-level localization.

In practice, after a one-time camera marker calibration with four fiducials attached to the torch, three non-collinear marks define an orthonormal torch-centric frame, and the fixed geometric offset from this frame to the physical nozzle tip is obtained. During operation, the camera repeatedly re-detects the three marks to recover the current pose of the torch frame with respect to the camera and transforms the pre-calibrated nozzle offset into the camera frame to recover the 3D tip coordinates in real-time. The estimated tip is then projected onto the workpiece plane and matched against the hole layout so that the UI immediately highlights the index of the hole just welded. Under harsh conditions, the YOLO–LaserGalvo module is engaged to place an IR spot at the desired pixel and acquire a metric distance, thereby resolving monocular scale ambiguity and improving robustness while preserving the same pose estimation and visualization flow.

### 3.2. Monocular Vision Fundamentals and Fusion with Infrared Ranging

**Figure 2:**
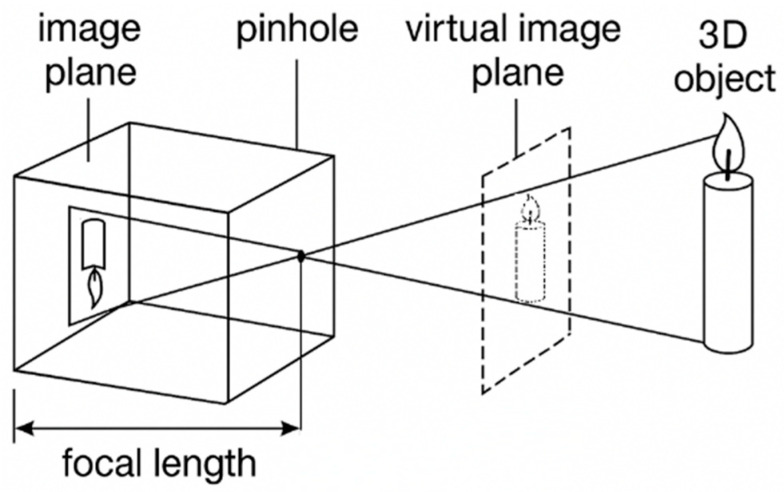
Schematic of the pinhole camera imaging model.

A monocular vision system is commonly modeled on the ideal pinhole camera as illustrated in Figure 2. Let a spatial point P(X,Y,Z) lie in the camera coordinate system; after passing through the pinhole at focal length f, its image is formed at p(u,v) on the image plane. The perspective projection satisfies:

(1) $$u=f\frac{X}{Z},\;v=f\frac{Y}{Z}$$

which can be written in homogeneous form as

(2) $$s\left[\begin{array}{c} u\\ v\\ 1 \end{array}\right]=K\left[\begin{array}{cc} R & t\\ 0 & 1 \end{array}\right]\left[\begin{array}{c} X\\ Y\\ Z\\ 1 \end{array}\right]$$

K denotes the intrinsic matrix, R,t the extrinsic parameters, and s represents the scale factor. This model reveals one-to-one projective mapping between image and spatial coordinates: the depth Z governs the scene’s scaling on the image plane, while a single image provides only relative depth, leaving the absolute scale undetermined. In this study, an infrared laser rangefinder supplies the true target-to-camera distance; the measured Z is fused with the projection model to recover accurate 3D coordinates in the camera frame, thereby enabling millimeter-level spatial accuracy for downstream industrial welding localization. To ensure geometric consistency, radial and tangential lens distortions are calibrated and corrected [26,27]. The matrix K is obtained via Zhang’s calibration method, with the reprojection error controlled within 0.2 pixel.

Equation (2) makes explicit the one-to-one projective mapping between image and spatial coordinates: the depth Z regulates the scale on the image plane, whereas a single view yields only relative depth and thus suffers from absolute scale ambiguity. To eliminate this ambiguity, we introduce an IR laser rangefinder to obtain the true target-to-camera distance. Substituting $Z_{\mathrm{IR}}$ into (1) and back-projecting directly recovers the target’s $\left(X,Y,Z_{\mathrm{IR}}\right)$ in the camera coordinate system, forming a depth–pose closed loop. First order error-propagation analysis indicates that after fusion, the depth standard deviation is about 0.5% Z, and the lateral error scales linearly with $\sigma_{Z}$. Compared with approaches relying solely on stereo or structured light, laser ranging maintains <1 mm fluctuation even under welding fumes.

**Figure 3:**
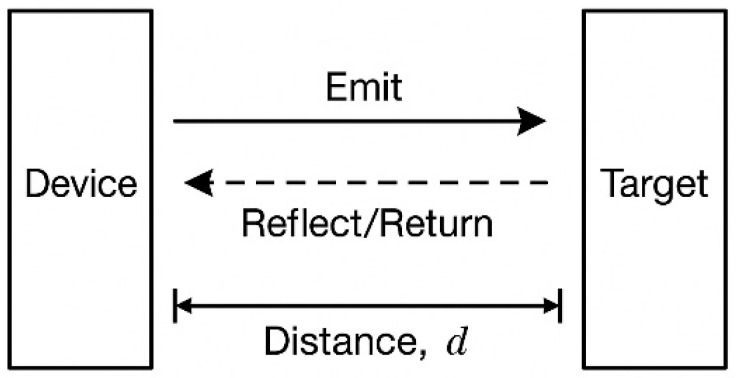
Schematic of the active distance measurement principle.

Infrared laser ranging provides an effective complement to monocular vision for depth compensation. IR ranging methods include laser triangulation and time-of-flight (ToF). Laser triangulation is shown in Figure 3 which exploits the geometric triangle formed by the emitter, the target, and the imaging receiver [28,29].

Let the baseline between the emitter and the receiving optics be b, and the focal length of the receiving lens be f. When the laser spot reflected from the target forms an image on the sensor, its image plane offset relative to the optical axis is Δx. By the principle of similar triangles, the target range d satisfies $d=\frac{bf}{\Delta x}$.

Alternatively, if the optical geometry is parameterized by the angle θ between the received ray and the baseline, with $\tan\theta =\frac{\Delta x}{f}$, a closed-form range expression can likewise be derived as $d=\frac{b}{tan\theta }$.

Time-of-flight ranging computes distance by measuring the round-trip propagation time t of a light pulse. Given the constant speed of light c, the range can be obtained from $d=\frac{ct}{2}$. ToF ranging requires minimal calibration and supports long-range measurements, which is why it is widely used in LiDAR and large-scale scene scanning. However, its accuracy can be affected by instrument time-base drift and by the target’s reflectance.

**Figure 4:**
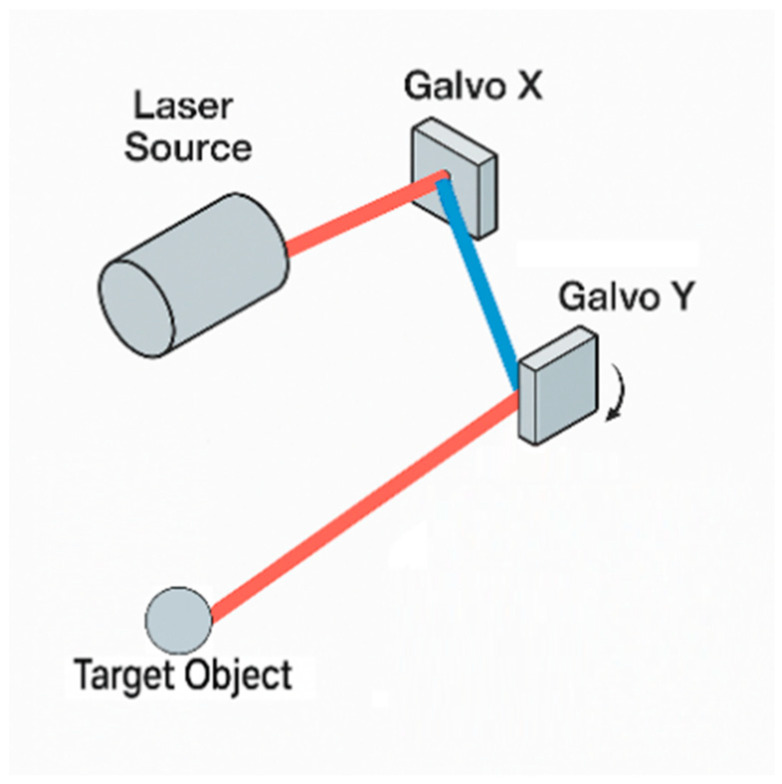
Dual-axis laser galvanometer scanning system.

**Figure 5:**
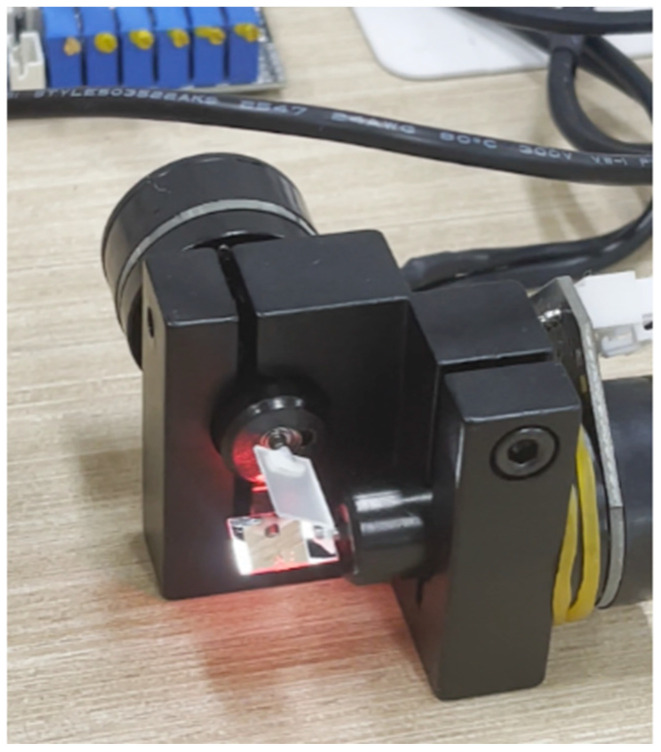
The galvanometer assembly employed in our experiments.

A galvanometer is an oscillatory electromagnetic actuator. When current flows through its coil, a torque is generated in the magnetic field that deflects the mirror against a return spring; to first order, the deflection angle is proportional to the drive current. Precise current control therefore enables real-time control of mirror tilt. A two-dimensional galvo comprises two orthogonal single-axis scanners (X and Y) that steer the laser horizontally and vertically, enabling rapid rastering of the target plane and millisecond-level precision scanning; a schematic is shown in Figure 4, and the experimental galvo assembly is shown in Figure 5.

To facilitate tracking a moving fiducial, the dual-axis galvo scan head is mounted orthogonally to the infrared rangefinder. The galvo comprises two mirrors arranged in orthogonal axes: a front *X*-axis mirror performs fast rastering, while a rear *Y*-axis mirror provides slower deflection; together they determine the 2D impact point of the laser beam on the workpiece plane. When an f-θ lens is used to simplify the optical path, one obtains:

(3) $$X=f\tan\theta_{x},\;Y=f\tan\theta_{y}$$

where f is the effective focal length of the lens and $\theta_{x},\theta_{y}$ are the mechanical deflection angles of the two axes. This compact geometric model underpins subsequent distortion compensation and trajectory planning. When the working plane is tilted by α relative to the camera optical axis, a rotation matrix should be introduced to transform between the workpiece and camera coordinate frames so as to preserve coordinate consistency, i.e., $R_{cw}$.

(4) $$G(s)=\frac{K}{\tau^{2}s^{2}+2\zeta \tau s+1}$$

The galvo actuation dynamics can be characterized by a standard second-order form, where K denotes the gain, τ the electromechanical time constant, and ζ the damping ratio. High-end galvos can achieve a 10–90% rise time below 250 μs under ±0.1° small step inputs, corresponding to a −3 dB bandwidth up to 1.5 kHz. A holistic evaluation indicates that the maximum vector-marking (fly-marking) linear speed reaches 3–5 $ms^{-1}$ while maintaining a roundness error of 25 μs.

In summary, IR ranging resolves the scale ambiguity inherent in monocular vision, delivering millimeter-level accuracy at meter-scale working distances. The fused reconstruction algorithm jointly estimates depth and pose, while the galvo scanner’s sub-millisecond response enables rapid pointing and micro-positioning; these capabilities are critical for weld-path detection and closed loop control.

### 3.3. Improved YOLOv11 Object Detection Network

**Figure 6:**
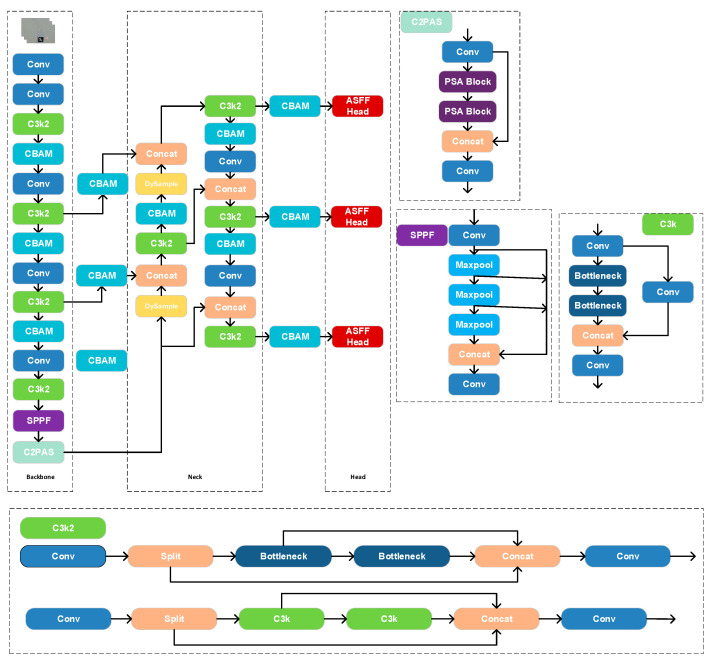
Schematic of the enhanced YOLOv11 architecture.

To enhance the feature expressiveness and small-object detection of YOLOv11, we co-optimize lightweight efficiency and accuracy within the original framework so that the model sustains high recall and real-time throughput in complex industrial scenes with welding fumes. Three modifications are introduced to the Backbone, Neck, and Head, as illustrated in Figure 6. In the Backbone, a Convolutional Block Attention Module (CBAM) is inserted to strengthen channel- and spatial-wise representation. In the Neck, the upsampling stages of the feature pyramid are replaced with DySample to recover finer details. Finally, in the Detection Head, an Adaptive Spatial Feature Fusion Head substitutes the original head to adaptively fuse multi-scale feature maps and reinforce target representation.

**Figure 7:**
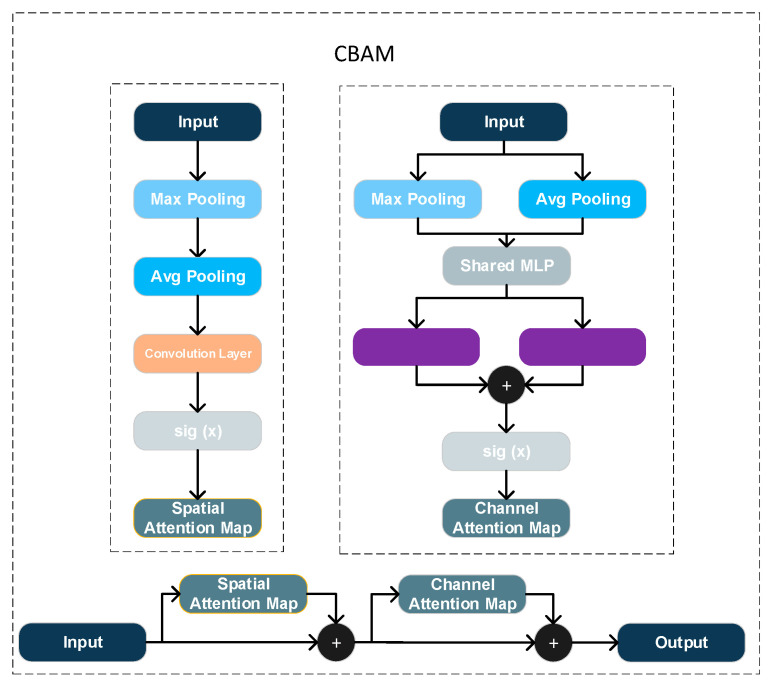
Diagram of the CBAM (Convolutional Block Attention Module).

In the Backbone feature extractor, a CBAM block is inserted after the output of the C3k2 module to improve feature selectivity. As Figure 7 shows, CBAM consists of sequential channel and spatial attention; it first derives per-channel global descriptors via global average and max pooling and then computes channel weights through fully connected layers; subsequently, a convolution produces a spatial attention map. The resulting channel and spatial masks are applied to the input features via element-wise multiplication, thereby emphasizing informative responses and suppressing redundancy [30]. Compared with the original YOLOv11 without attention, CBAM guides the network to focus on more discriminative channels and regions, improving the localization and discrimination of small targets [31]. Because CBAM adds only a small number of parameters and relies on simple operations such as global pooling and convolutions, its impact on inference speed is negligible.

**Figure 8:**
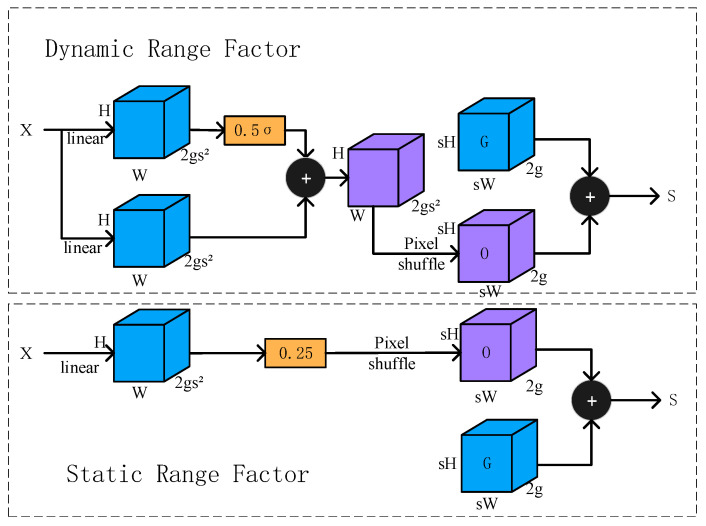
Illustration of the DySample dynamic sampling module.

For the Neck, we improve the top-down feature pyramid fusion by replacing two upsampling operations with DySample. The nearest neighbor upsampling used in vanilla YOLOv11 employs a fixed interpolation scheme that may cause detail loss and checkerboard artifacts. As Figure 8 shows, DySample is a dynamic upsampling method that learns sampling locations conditioned on the input features, thereby producing higher-quality upsampled maps. Concretely, a lightweight subnetwork predicts sampling offsets for each output location, and interpolation is performed on the low-resolution feature map according to these offsets [32]. With minimal computational overhead, DySample effectively increases the fidelity of the upsampled features, reconstructing more details and textures that benefit subsequent fusion with low-level features and the recognition of small objects.

In the Detection Head, we replace the original head with the Adaptive Spatial Feature Fusion (ASFF) Head to achieve adaptive fusion and strengthened expression across scales. In the standard YOLOv11 head, classification and regression are performed independently on each scale, with only limited cross-scale interaction provided by FPN/PAN structures in the Neck. ASFF enables dynamic fusion of multi-scale features at the output; for each detection scale, features from other scales are first up/downsampled to a common resolution and unified in channel number via 1 × 1 convolutions; they are then fused with the native features through pixel-wise weighting, where the weights are learned by a lightweight subnetwork based on the responses of different scales [33]. This adaptive scheme allows the network to select, at each spatial location, the most informative scale while suppressing irrelevant or conflicting information from other scales, thereby mitigating the inconsistency among pyramid levels. ASFFHead introduces only a few 1 × 1 convolutions and linear weighting operations, rendering the additional computation negligible; compared with the original head, it significantly improves the utilization of multi-scale information and boosts detection performance across object sizes [34]. For small-object detection in particular, ASFFHead combines the fine details from high-resolution features with the semantic context from low-resolution features, markedly reducing misses and improving localization accuracy.

### 3.4. Marker Detection and Pose Estimation Methods

**Figure 9:**
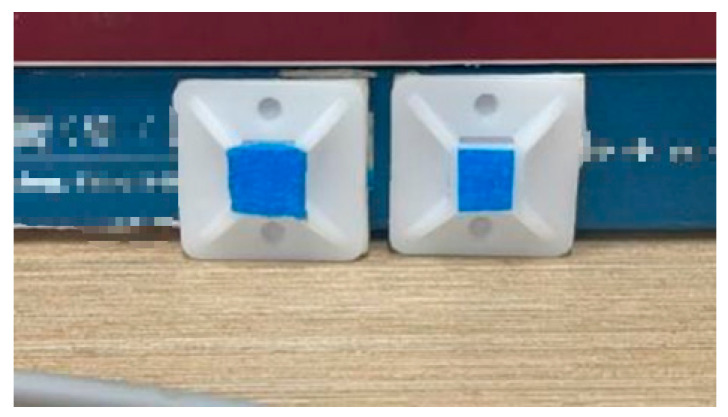
Color-marker positioning stickers.

**Figure 10:**
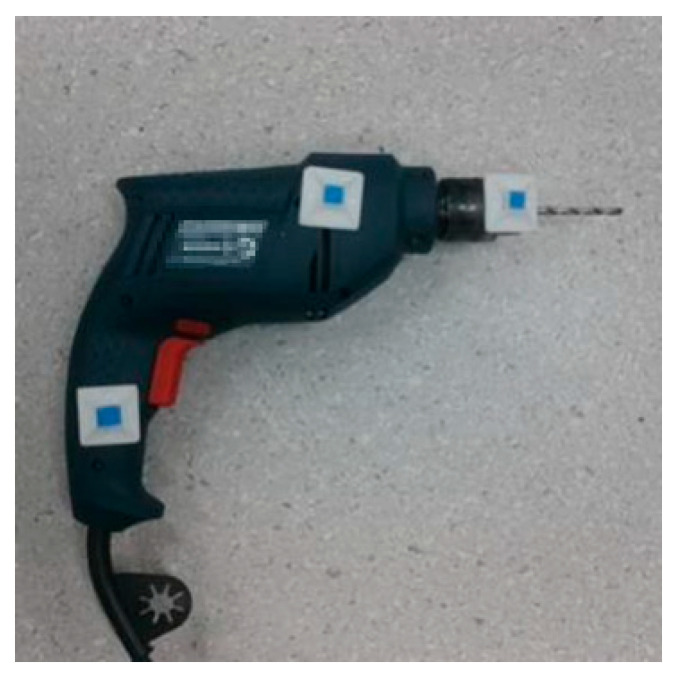
Usage of color-marker positioning stickers.

**Figure 11:**
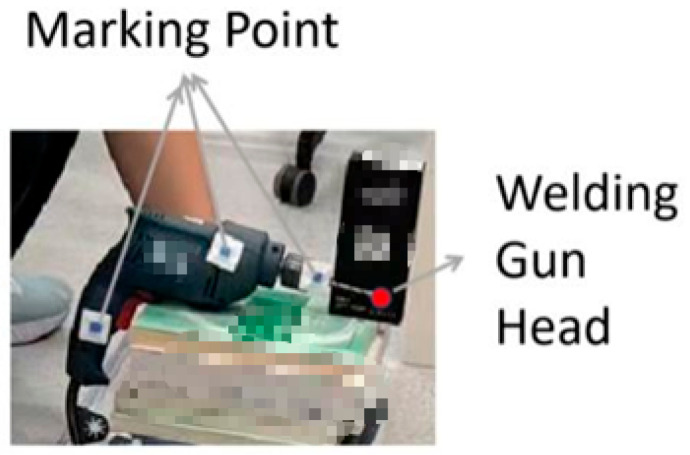
Welding gun calibration, physical diagram.

To precisely localize the welding torch in 3D space, this study advances the detection of fiducials on the torch tip from simple to more sophisticated techniques. As Figure 9, Figure 10 and Figure 11 shows, we first adopt a color-marker approach: a sticker of a specific hue (e.g., blue) is affixed to the torch surface and detected via computer vision color segmentation.

In implementation, the RGB image is converted to HSV space; a preset hue range is applied for binary segmentation of the target color to produce a mask of the marker region. Morphological opening/closing removes noise, after which connected-component analysis extracts the region and the centroid is computed to obtain the image coordinates of the mark:

(5) $$Mask(x,y)=\left\{\begin{array}{ll} 1, & \mathrm{if}\;HSV(x,y)\in [lower\_blue,upper\_blue]\\ 0, & \mathrm{otherwise} \end{array}\right.$$

The method is conceptually simple and real-time, performing well in laboratory settings with uniform illumination and minimal background clutter. However, because it relies solely on color information, its robustness is limited, and changes in ambient lighting or the presence of similarly colored backgrounds can degrade accuracy. Consequently, a fixed-threshold scheme is ill-suited to field conditions without illumination compensation or dynamic thresholding, reducing engineering applicability.

**Figure 12:**
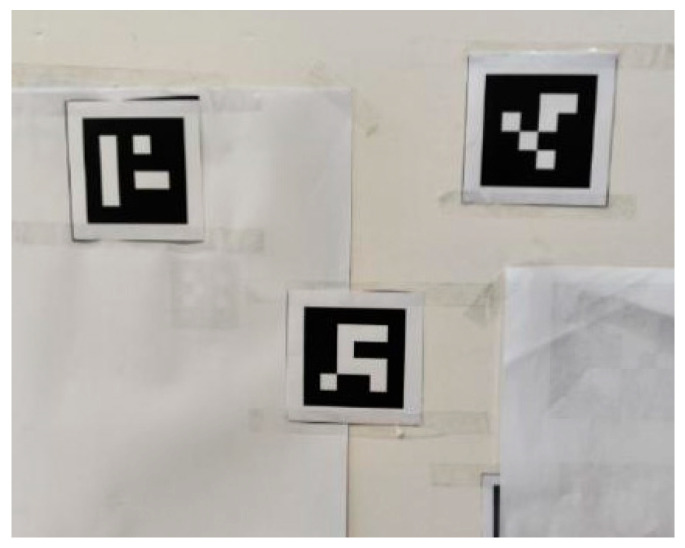
ArUco Shape Marker Points.

To improve robustness, we introduce ArUco-based detection. As Figure 12 shows, an ArUco marker is a planar, binary-coded fiducial with a unique ID that can be quickly localized through geometric pattern recognition.

**Figure 13:**
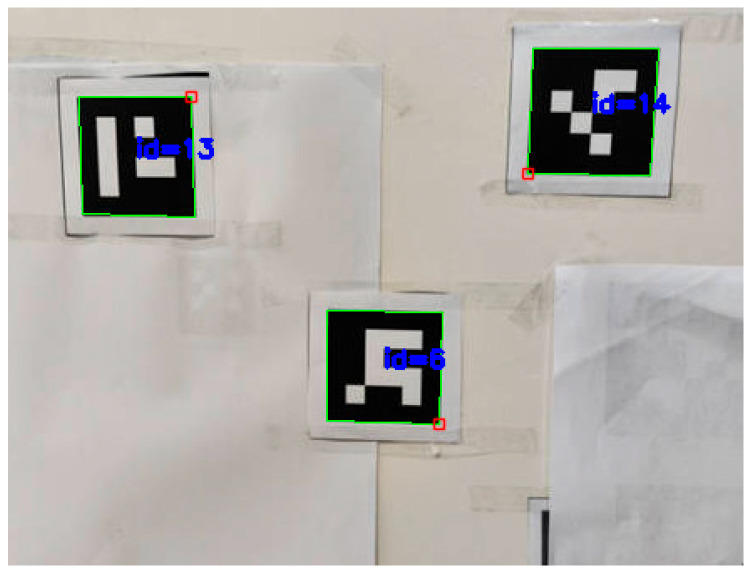
ArUco marker recognition.

During detection, the image is first converted to grayscale to reduce computation; OpenCV’s ArUco library then identifies the marker and its corner coordinates. The marker center is obtained by averaging the four corners, yielding the image coordinates of the mark. Furthermore, given the known physical size of the ArUco code and the camera intrinsics, a PnP solution provides the marker pose relative to the camera, and from the four corner projections one estimates the rotation matrix R and translation vector t, i.e., the extrinsic matrix T=[R|t], enabling accurate 3D localization of the mark. Compared with color thresholding, the ArUco method is more robust to illumination and background variations and reliably recognizes the marker and estimates its position even under uneven lighting or clutter. Its unique ID also facilitates multi-marker association and tracking (in Figure 13).

This approach, however, requires printed codes attached to the torch, which may be contaminated by weld spatter in practice; the recognition pipeline is also more complex than color segmentation and consumes additional compute. Overall, ArUco-based detection outperforms color thresholding in accuracy and stability, making it better suited to in situ torch-pose estimation.

**Figure 14:**
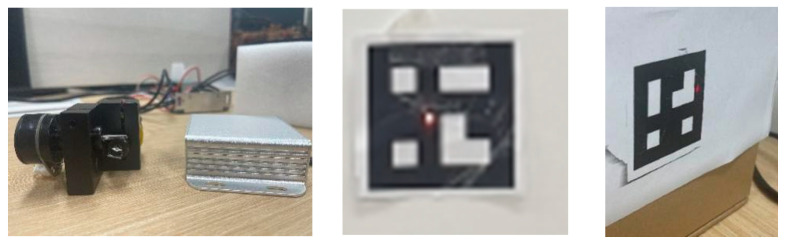
Experimental setup and laser aiming example. (**Left**) The laser galvanometer assembly alongside the laser range-finding module; (**Center**) red laser spot projected onto the ArUco calibration target; (**Right**) view of the target affixed in the scene.

Building on these methods, we further propose an active mark-detection scheme that combines deep learning object detection with an infrared range sensor to achieve intelligent mark recognition and high-precision depth/pose measurement. To address the susceptibility of passive visual markers in complex environments and the limited depth accuracy of purely visual ranging, the system integrates an improved YOLOv11 model with an active IR laser-ranging unit, thereby realizing a 3D localization method that couples laser-based active marking, intelligent detection, and precise ranging. The core idea is to project an infrared laser spot as an active fiducial onto the target surface; the camera captures the spot and YOLOv11 detects it to obtain its pixel coordinates. In parallel, the image location of the region of interest (e.g., the center of an ArUco code on the torch to be calibrated) is determined by the preceding method. The pixel offset of the IR spot relative to the target point (horizontal and vertical differences) is computed and mapped to galvo control inputs to steer the spot to the target in real-time (Figure 14). Finally, the IR rangefinder measures the true distance at the target point. By pairing the target’s image coordinates with the measured range and invoking the camera imaging model, the 3D coordinates of the target point are computed. For example, in the camera frame, if the target’s pixel coordinates are u_target , v_target, the camera intrinsics are $\left(f_{x},f_{y},c_{x},c_{y}\right)$, and the measured distance is D, then the spatial coordinates of the point are

(6) $$X=\frac{(u_{target}-c_{x})D}{f_{x}},\;Y=\frac{(v_{target}-c_{y})D}{f_{y}},\;Z=D$$

Driven by the galvo, the IR sensor is directed to the YOLOv11-detected region, the distance there is measured, and the 3D coordinates of the mark are obtained by fusing range with image coordinates. Compared with stereo-based visual ranging, the IR sensor provides a direct distance measurement, avoiding disparity-matching errors; meanwhile, YOLOv11’s learned features confer robustness under severe illumination changes and welding fumes. This deep learning + sensor-fusion approach offers high accuracy, fast response, and strong engineering viability. On one hand, YOLOv11 can be trained and optimized to meet industrial requirements for detection accuracy and real-time performance; on the other hand, IR ranging is independent of ambient light and supplies stable depth in bright-arc or dim welding environments. In sum, YOLOv11-based recognition fused with IR ranging substantially improves the robustness and accuracy of torch-pose perception and is well suited for deployment in practice.

### 3.5. Three-Dimensional Localization and Coordinate Transformation

This section establishes a torch-centric coordinate frame from three detected fiducials and derives the welding-nozzle position, followed by a spatial re-localization method. During calibration, three non-collinear marks pre-attached to the torch define the torch coordinate frame. Let their coordinates in the global frame be $A\left(x_{1}y_{1},z_{1}\right)$, $A\left(x_{1}y_{1},z_{1}\right)$, and $C\left(x_{3}y_{3},z_{3}\right)$. The frame is computed as follows:

1. Reference vectors: Taking A as the reference point, compute two direction vectors $\vec{AB}$ and $\vec{AC}$ as $\vec{AB}=(x_{2}-x_{1},y_{2}-y_{1},z_{2}-z_{1})$ and $\vec{AC}=(x_{3}-x_{1},y_{3}-y_{1},z_{3}-z_{1})$, respectively. These vectors span the principal plane of the torch frame.
2. Orthogonal axes construction: Using $\vec{AB}$ and $\vec{AC}$, construct three mutually orthogonal unit basis vectors according to the right-hand rule, forming the three axes of the torch frame. For example, set $e_{1}$ as the unit vector of $\vec{AB}$; then compute $e_{3}=\frac{\vec{AB}\times \vec{AC}}{\left||\vec{AB}\times \vec{AC}|\right|}$ as the unit normal to the plane spanned by AB; finally, let $e_{2}=e_{3}\times e_{1}$ be the third unit vector orthogonal to the previous two. Thus $e_{1},e_{2},e_{3}$ forms the torch coordinate frame A with origin at R.
3. Measure the relative placement of the nozzle reference point (D) with respect to the three fiducials. First, determine the global coordinates of D (via stereo triangulation or other metrology). Then compute the vector from D to A, denoted $\vec{AD}=(x_{D}-x_{1},y_{D}-y_{1},z_{D}-z_{1})$, and project this vector onto the three axes of the torch frame R; the projection lengths are the coordinate components of D in the torch frame. Denote the nozzle D in the torch frame by G=(u,v,w); then $u=\vec{AD}\cdot e_{1}$, $v=\vec{AD}\cdot e_{2}$, $w=\vec{AD}\cdot e_{3}$. This completes the calibration of the nozzle relative to the fiducial-defined frame, where G represents the fixed nozzle position in the torch frame.

Having calibrated the nozzle position in the fiducial-defined torch frame, we can rapidly re-localize the nozzle in space when the torch moves. The core idea is to re-detect the three marks at the new pose, rebuild the torch frame, and transform the previously calibrated nozzle-relative coordinates G into the updated frame to obtain the actual spatial position $G^{\prime }$. The detailed steps are as follows:

4. Acquire fiducial coordinates at the new pose: After the torch moves, use vision to measure the three fiducials’ 3D coordinates, denoted $A^{\prime }({x_{1}}^{\prime },{y_{1}}^{\prime },{z_{1}}^{\prime })$, $B^{\prime }({x_{2}}^{\prime },{y_{2}}^{\prime },{z_{2}}^{\prime })$, $C^{\prime }({x_{3}}^{\prime },{y_{3}}^{\prime },{z_{3}}^{\prime })$.
5. New vector basis construction: Taking $A^{\prime }$ as the reference, similarly compute $\vec{A^{\prime }B^{\prime }}=({x_{2}}^{\prime }-{x_{1}}^{\prime },{y_{2}}^{\prime }-{y_{1}}^{\prime },{z_{2}}^{\prime }-{z_{1}}^{\prime })$ and $\vec{A^{\prime }C^{\prime }}=({x_{3}}^{\prime }-{x_{1}}^{\prime },{y_{3}}^{\prime }{y_{1}}^{\prime },{z_{3}}^{\prime }-{z_{1}}^{\prime })$, and from these constructs the unit basis vectors $R^{\prime }$ of the new coordinate frame ${e_{1}}^{\prime },{e_{2}}^{\prime },{e_{3}}^{\prime }$.
6. Solve the attitude transform: From the directional relations of the basis vectors in the old and new frames, estimate the rotation matrix R that maps the calibration-time torch frame $R^{\prime }$ to the current torch frame $R_{\mathrm{rel}}$. In practice, the column vectors of $R_{\mathrm{rel}}$ can be taken as the new basis vectors expressed in the old frame R, or equivalently one can obtain the transpose of $R_{\mathrm{rel}}$ from the old basis expressed in the new frame. In short, with the bases from Steps 1–2, construct the rotation describing the torch’s attitude change, i.e., $R_{\mathrm{rel}}\in {\mathbb{R}}^{3\times 3}$.
7. Re-localize the nozzle in space: Transform the calibrated nozzle-relative coordinates G=(u,v,w) with the above rotation, add the translation $A^{\prime }$ (the new frame origin in global coordinates), and obtain the nozzle’s new global position $G^{\prime }$. This relation can be written as $G^{\prime }=R_{\mathrm{rel}}\cdot G+\vec{OA^{\prime }}$, where $\vec{OA^{\prime }}$ denotes the position vector of fiducial $A^{\prime }$ in the original global frame. Thus, 3D positioning of the nozzle under arbitrary pose changes is achieved, completing the re-localization.

Using this method, the torch frame is established from three known fiducials and the nozzle position is calibrated once; upon motion, it suffices to remeasure the fiducials to rapidly compute the nozzle’s updated 3D coordinates. This vector-projection and coordinate-transform approach avoids repeated direct measurements on the nozzle and offers high localization accuracy and computational efficiency.

### 3.6. Real-Time 3D Localization, Visualization, and System Implementation

The proposed system integrates the foregoing algorithms into a unified hardware–software architecture to realize real-time 3D localization and visualization during welding. The hardware stack comprises a stereo vision sensor, an infrared range unit, and a controllable dual-axis galvo; the software stack combines deep learning detection, image processing, and 3D computation. The overall workflow is as follows: the camera first localizes the torch fiducials or other targets in pixel coordinates. Next, a YOLOv11 model detects the laser spot in the acquired image; the system drives the IR range sensor via the galvo to the target region to obtain the depth value, which is then fused in the camera coordinate frame to compute the target’s 3D coordinates. Meanwhile, precise galvo control enables the range sensor to rapidly refocus on regions of interest, supporting sequential measurements of multiple points. The ranging–detection loop is fully automated and maintains high efficiency and robustness under welding conditions.

To address dynamic environmental changes during welding, a luminance-change trigger is designed: the system continuously monitors scene brightness and automatically launches image capture and depth measurement when the global intensity change induced by the welding arc exceeds a preset threshold. This trigger is sensitive and real-time, ensuring timely data acquisition at critical instants. A debounce strategy—minimum interval and delay—is employed to avoid excessive triggers due to environmental noise or device adjustment. In sum, the integrated trigger-and-acquisition mechanism ensures reliable operation in complex environments.

For efficient and accurate 3D computation, ROI restriction and threshold filtering are applied. Based on the YOLOv11 target location, a local ROI is cropped in the depth map so that distance estimation focuses only on depths near the target, substantially reducing irrelevant computation and improving throughput. Valid depth bounds are enforced to remove outliers; for example, only measurements within 100–10,000 mm are accepted. Nearer-than-lower-bound or beyond-upper-bound readings are typically unreliable or outside the sensor’s accuracy range and are rejected to prevent contamination of localization results.

To ensure accuracy of the IR range sensor, a calibration procedure is performed: a set of known distances is measured to derive the mapping between sensor readings and ground truth distance. Linear fitting determines correction parameters, and the raw readings are rectified accordingly. For instance, the linear correction formula $Z_{\mathrm{actual}}=k\cdot Z_{\mathrm{meas}}+b$ is applied to the sensor reading $Z_{\mathrm{meas}}$ to obtain the corrected distance $Z_{\mathrm{actual}}$, which better approximates the true value. With ROI selection, threshold filtering, and calibration, the system yields stable and reliable 3D coordinates.

The camera intrinsics are then used to fuse pixel coordinates with depth to obtain the target’s 3D coordinates (X,Y,Z) in the camera frame. Here Z denotes the depth from the range sensor, and (X,Y) is derived from the imaging model using the pixel coordinates. For a pixel (u,v), its spatial position is computed from $X=\frac{(u-c_{x})Z}{f_{x}}$ and $Y=\frac{(v-c_{y})Z}{f_{y}}$ (with camera principal point $c_{x},c_{y}$ and focal length $f_{x},f_{y}$). Through this series of fusion steps, data from visual detection and range sensing are converted into 3D points in a unified coordinate system.

**Figure 15:**
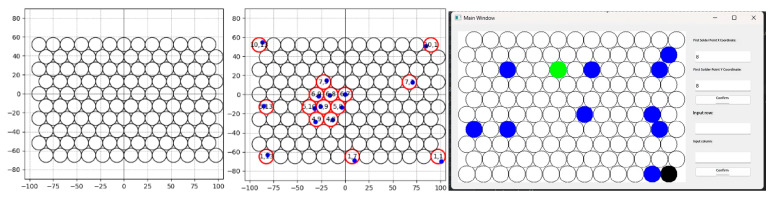
Visualization and interactive interface for the circular-grid layout. (**Left**) Generated 2-D mock welding-plate pattern; (**center**) example showing measured points alongside their estimated counterparts; (**right**) GUI window for interactive control.

To present localization results and assist operation, the system provides real-time visualization of welding scenes and 2D projection with label-matching for multiple hole positions on tube sheets. Prior to operation, a mathematical model of the hole layout is established. Once multiple hole positions on the actual workpiece are acquired, their 3D coordinates are projected to the workpiece plane via coordinate transformation to extract 2D planar coordinates. A matching algorithm then pairs measured planar points with the modeled hole coordinates to identify correspondences. Each detected hole is mapped to its corresponding index in the layout model (i.e., its designated position number). The system then visualizes the result by either generating a numbered planar projection or overlaying labels on the live image (Figure 15). This allows operators to clearly see correspondences and order between detected holes and the design model, guiding welding to proceed in the prescribed sequence. The real-time 3D visualization and label-matching functions significantly enhance usability and intelligence, ensuring accurate and reliable data interpretation and decision-making in complex welding tasks.

## 4. Experiments

### 4.1. Experimental Environment and Datasets

All experiments were conducted on Ubuntu 22.04 using the PyTorch 1.13.1 deep learning framework. The software/hardware environment included Python 3.9, Intel (R) Core (TM) i9-13900H CPU, NVIDIA GeForce RTX 3090 GPU. Unless otherwise stated, the input resolution was fixed at 640 × 640. Mosaic data augmentation was enabled and disabled during the final 10 epochs; batch size = 8, epochs = 200; and the SGD optimizer was used with an initial learning rate of 0.01.

A single-class, self-compiled dataset was employed, comprising 2000 high-resolution RGB images with the target class “laser” (laser spot). Data collection covered a broad range of illumination conditions (indoor/outdoor) and background materials, including both ideal scenes with sharp, high-contrast spots and challenging scenes with strong reflections and pronounced noise, to improve robustness to real operating conditions. Experiments considered two glare conditions: flashlight-simulated arc glare in the laboratory and real welding arc glare in a factory environment. All samples were annotated by at least two raters experienced in vision metrology using LabelImg to draw bounding boxes.

The dataset was randomly split 8:2 into training and validation sets, and 5% of the training images were held out as an internal validation subset for early-stopping monitoring. To prevent data leakage, frames from the same capture sequence were grouped and assigned as a unit. The final dataset maintained balanced class distribution, a unified 640 × 640 resolution, and employed Mosaic, HSV color jitter, and random affine augmentations during training to further enrich diversity and robustness.

### 4.2. Evaluation Indicators

To quantitatively assess the system’s measurement accuracy, the following error calculation methods were primarily used:

Root Mean Square Error (RMSE): This metric reflects the impact of larger error points. The formula is

(7) $$RMSE\;=\;\sqrt{\frac{{\left(X_{m}\;-\;X_{t}\right)}^{2}\;+\;{\left(Y_{m}\;-\;Y_{t}\right)}^{2}\;+\;{\left(Z_{m}\;-\;Z_{t}\right)}^{2}}{3}}$$

Mean Absolute Error (MAE): This represents the average absolute value of all errors in three-dimensional space. The formula is

(8) $$MAE\;=\;\frac{\left|X_{m}\;-\;X_{t}|\;+\;|Y_{m}\;-\;Y_{t}|\;+\;|Z_{m}\;-\;Z_{t}\right|}{3}$$

For each distance stage (left, center, right), multiple measurements were recorded. The errors were calculated and averaged to systematically evaluate the measurement stability and accuracy of the system at different locations.

### 4.3. System Implementation and Integration

To validate the performance of different localization schemes, we implemented and tuned three systems: Scheme I (color-marker thresholding), Scheme II (ArUco markers), and Scheme III (YOLOv11-based Laser–Galvo integration). The workflow comprises two stages: calibration and measurement. In the calibration stage, fiducials on the target are captured to establish a reference coordinate frame; in the measurement stage, the system performs real-time detection and 3D computation of target points to verify accuracy and real-time performance.

#### 4.3.1. Calibration Procedure

We use a 4.81 mm focal length lens. Radial and tangential distortions were calibrated via Zhang’s method, yielding the camera intrinsics K and distortion coefficients with a 13 × 7 chessboard (25 mm squares); the mean reprojection error is <0.2 px. All images used for 3D computation and accuracy evaluation were undistorted using OpenCV functions.

As summarized in Table 1, depth accuracy is high at short-to-mid ranges. Up to 110 cm, the absolute error is ≤0.20 cm, with the center column as low as 0.003 cm. Beyond 120 cm, the error grows approximately linearly with distance at 150 cm, and errors reach 0.51–0.67 cm, with edge positions slightly worse than the center. Overall, after calibration and undistortion, the system attains sub-millimeter to sub-centimeter accuracy at near ranges and maintains acceptable accuracy at 1.5 m, with the center of the field of view performing best.

**Figure 16:**
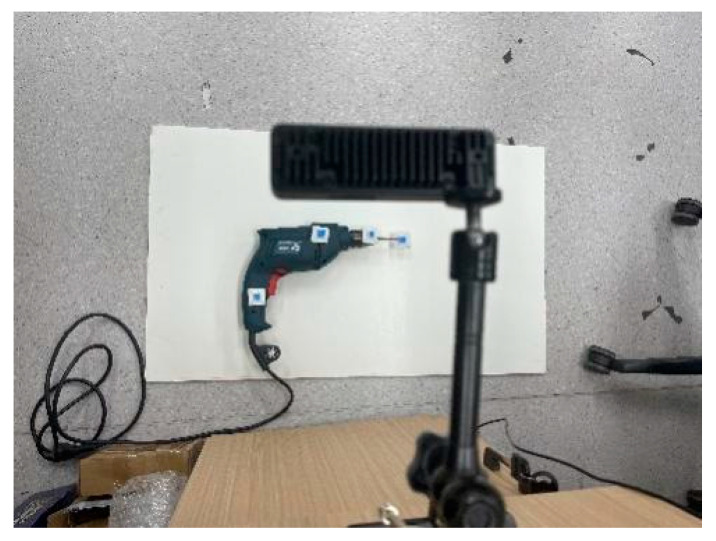
Color-marker calibration 1.

**Figure 17:**
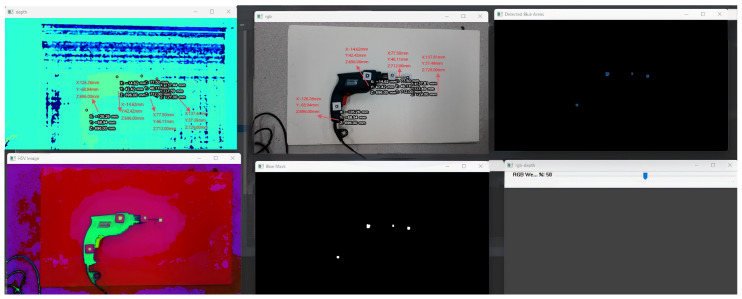
Color-marker calibration result.

**Figure 18:**
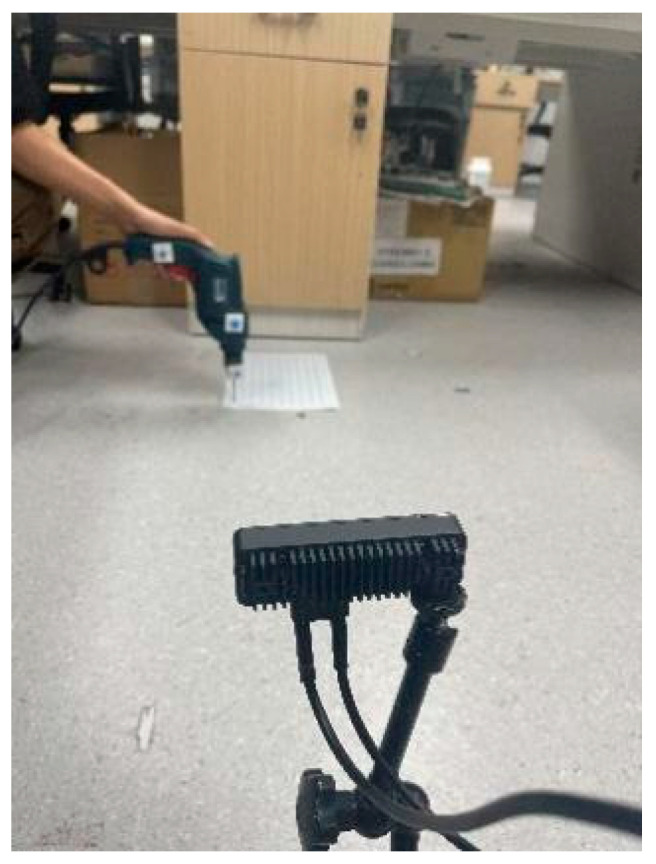
Color-marker measurement.

For the color-marker scheme, four blue stickers were attached around the mock torch tip (Figure 16). After stereo acquisition, the calibration routine estimated the torch end in the camera frame (Figure 17). For example, in one run the calibrated torch tip coordinates were [133.674, −104.902, 808.526] (camera frame). The result is saved (e.g., G_GUN.txt) for subsequent measurements. Figure 18 illustrates the simulated measurement scenario using color-markers.

For the ArUco marker scheme, a carton/plate was used to emulate the torch, with four ArUco tags affixed. Computer vision routines detected each marker’s four corners and pose, thereby completing camera frame calibration. Owing to their coded structure, ArUco markers are more tolerant to illumination and viewpoint changes than color thresholding, making calibration more stable and reliable.

For the YOLO–LaserGalvo scheme (Scheme III), an IR laser emitter was mounted near the camera and a rotary galvo steered the beam in space. During calibration, the torch was fixed at a known pose and the laser was triggered to project onto a reference plane. The YOLOv11 network detected the IR spot in real-time and yielded its pixel coordinates while the corresponding galvo control signals were recorded. From key-point measurements, a mapping was established between the laser spot’s 3D position in the camera frame and the galvo control inputs. After calibration, the laser spot’s 3D position in the reference frame was saved for closed loop operation. Benefiting from high-GPU inference rates and high-frequency galvo response, detection and control form a feedback loop: detections are fed back to the controller to adjust the beam direction in real-time, enabling target tracking.

#### 4.3.2. Ablation Study on YOLOv11 Improvements

**Figure 19:**
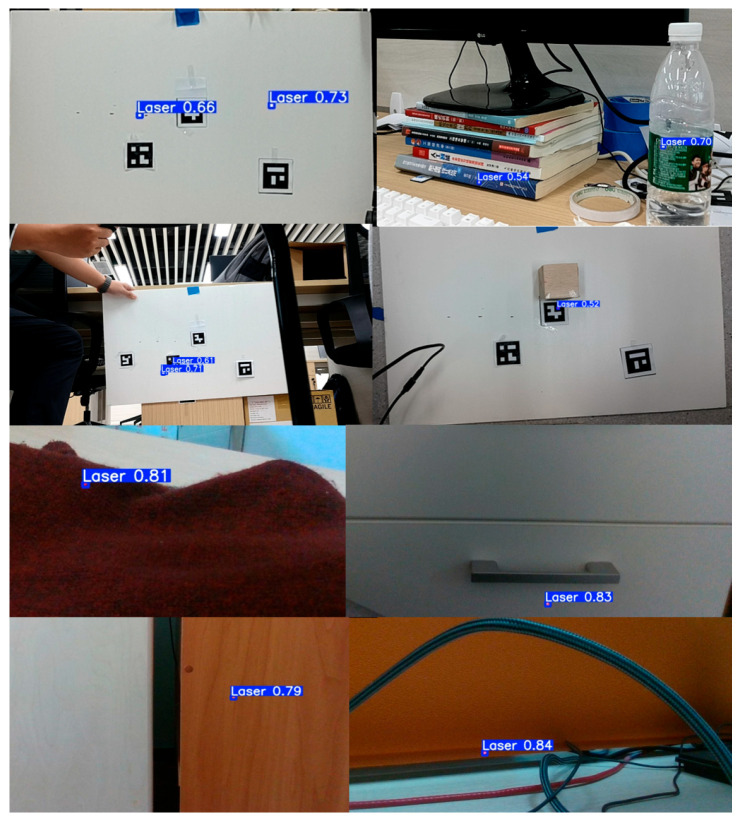
Example results of laser spot detection in various scenes.

As summarized in Table 2, we performed stepwise ablation on the YOLOv11 baseline. Adding CBAM alone yielded coordinated gains—Precision +1.3%, Recall +0.9%, mAP@0.5 +1.6%, mAP@0.5:0.95 +1.8%—indicating that channel–spatial attention helps suppress false positives from bright backgrounds. Building on CBAM, introducing DySample further increased mAP@0.5 to 75.5% (an additional +0.7% over CBAM only), while mAP@0.5:0.95 decreased slightly (−0.5%), suggesting that stronger multi-scale upsampling benefits coarse IoU matching (IoU = 0.5) but may slightly weaken boundary adherence at high IoU. Finally, adding ASFFHead on top of CBAM + DySample achieved the best performance on all four metrics—Precision 70.3%, Recall 72.8%, mAP@0.5 76.8%, mAP@0.5:0.95 46.3%—which improved over the YOLOv11 baseline by +2.9%/+3.0%/+3.6%/+2.7%, with reduced oscillation in training/validation curves. This indicates that adaptive multi-scale fusion maintains recall while improving high-IoU boundary quality, yielding more stable convergence and higher overall accuracy. Figure 19 illustrates results of laser spot detection in different scenes.

#### 4.3.3. Real-Time Measurement and Visualization

After calibration, a planar schematic of the tube-sheet was placed approximately 1.2 m from the camera, and measurements were taken with the calibrated torch. The system automatically detected blue markers in the image, computed their 3D positions, and overlaid the estimated torch tip position onto the measurement interface. With repeated torch movements, the system output the tip’s position changes in real-time, confirming the feasibility of the color-marker scheme under stable illumination.

The workflow mirrors the color-marker case. The system detects ArUco tags in the camera view and recovers their 3D poses to compute the torch end position. In experiments, the tube-sheet schematic was similarly placed, and the calibrated torch performed multi-point measurements. Compared with color thresholding, the ArUco method remained more stable during detection; even with tag rotations or complex backgrounds, the system accurately captured target positions.

**Figure 20:**
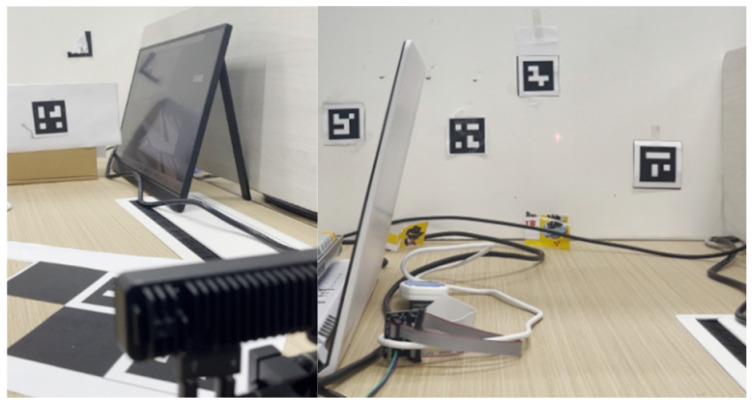
Experimental scene of the YOLOv11-based laser–galvo integrated system.

In Scheme III, the controller first projects an IR laser spot toward the target via the galvo (Figure 20). After image capture, YOLOv11 detects the spot location in real-time. The detected pixel coordinates are combined with the galvo’s angular control signals and, using the pre-established calibration mapping, the spot’s 3D coordinates are computed. In parallel, the built-in IR range sensor measures the distance from the spot to the torch and returns it to the computation module. Fusing pixel localization with actual distance measurements, the system updates and displays the spot’s 3D position in real-time. For closed loop control, the controller adjusts the galvo according to the error between the spot and the target, keeping the beam locked on the desired point. This scheme exploits YOLO’s high-speed detection and the galvo’s rapid response to realize a highly real-time, strongly closed loop measurement process.

### 4.4. Accuracy Verification and Error Analysis

We evaluate the localization accuracy of three schemes—color-marker thresholding, ArUco markers, and the YOLOv11-based laser–galvo integration—via repeated experiments and simulations and analyze potential error sources. We focus on near-field operation (0.9–1.5 m), assessing measurement stability and robustness differences under varying environmental conditions.

(1) Stereo camera-ranging accuracy: An OAK stereo camera (resolution 800p, baseline 75 mm) was used for depth sensing. After calibration, depth accuracy remained high over 0–4 m; measured range errors stayed within 2% in this span. This suffices for precise near-field (<2 m) localization and underpins subsequent error analyses.

(2) Color-marker accuracy and error analysis: The method converts RGB to HSV and thresholds blue stickers. Under stable lab lighting it is fast and simple; however, illumination/background variations (field-like conditions) significantly degrade recognition. Table 3 reports error statistics at different ranges. Both RMSE and MAE grow nonlinearly with distance, e.g., at 1.5 m, RMSE 13.54 mm and the max error reaches 17.20 mm, indicating high sensitivity to color/lighting shifts. Overall, the color method suits controlled labs but struggles to meet high accuracy demands in complex scenes.

(3) Accuracy and error analysis of the ArUco marker method. Compared with the color-marker approach, the ArUco method delivers higher detection accuracy and environmental adaptability. By estimating 3D pose from the four corners and rotation of each tag, it is less sensitive to single-hue or illumination changes. Experiments over the 0.9–1.5 m range show average RMSE = 6.24 mm and MAE = 5.85 mm, both markedly lower than the color method; the maximum error at 1.5 m is only 11.12 mm in Table 4. These error levels indicate that ArUco maintains strong stability across complex scenes, including high-contrast lighting and cluttered backgrounds. Overall, although it is slightly more computationally demanding and marginally slower, its robustness and accuracy make it well suited for high-precision applications in industrial automation and robotic localization.

(4) Accuracy and error analysis of the YOLOv11 Laser–Galvo Scheme: To assess Scheme III, we conducted simulated error analysis over the 0.9–1.5 m range under the same conditions. As summarized in Table 5, the YOLO–LaserGalvo (YLGS) system achieves markedly better accuracy than the color and ArUco methods: the average RMSE is 4.76 mm and the average MAE is 4.43 mm, whereas the average RMSEs of the color and ArUco methods in the same range are 8.59 mm and 6.24 mm, respectively, in Table 5. Errors increase only slowly with distance—RMSE 3.19 mm at 0.9 m rising to 6.79 mm at 1.5 m—demonstrating robustness for short-to-mid-range welding scenarios.

### 4.5. System-Level Evaluation

**Figure 21:**
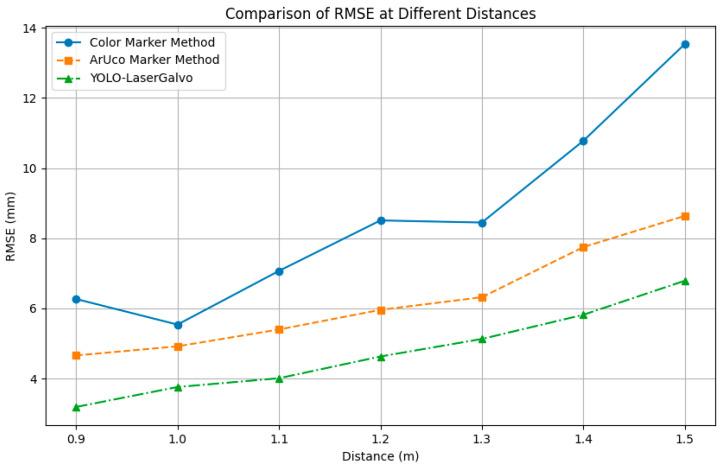
Comparison of RMSE at different distances between the three methods.

Across the 0.9–1.5 m range, errors increase with distance for all three schemes (Figure 21).

The YOLO–LaserGalvo approach consistently achieves the lowest RMSE/MAE with the mildest slope and the smallest fluctuations, indicating superior accuracy and stability. ArUco shows intermediate performance with an approximately linear increase, whereas the color-marker method exhibits the largest errors and a pronounced surge beyond 1.3 m, implying higher distance sensitivity and variability. In terms of RMSE, YOLO–LaserGalvo is typically ~40–50% lower than the color-marker method and ~20–30% lower than ArUco across most distances.

## 5. Discussion

### 5.1. Performance Profiling Under Welding Condition

Within the 0.9–1.5 m interval, the ranging error of all three schemes increases with distance (Figure 21), yet their absolute error levels and variability differ markedly. Statistics show that the YOLO–LaserGalvo scheme achieves the lowest RMSE and MAE values, indicating clear advantages in both accuracy and stability; the color-marker scheme produces the largest errors and highest MAE values, reflecting greater fluctuation; and ArUco resides in between with moderate overall performance.

In terms of real-time performance, the YOLO–LaserGalvo scheme exhibits a pronounced advantage. With GPU-accelerated YOLOv11 inference, throughput is high; for example, YOLOv11-n yields ~5.86 ms per frame (≈170 fps), vastly exceeding typical CPU-bound pipelines. The resulting low latency and high frame rate support responsive operation in dynamic measurement scenarios, whereas the color and ArUco pipelines, being primarily CPU-limited, generally run at lower frame rates and are less suitable for latency-sensitive tasks.

Robustness also differs across methods. As a data-driven model, YOLOv11 adapts well to cluttered backgrounds and moderate illumination shifts, though extreme lighting and heavy occlusion can still degrade detection. ArUco—relying on high-contrast fiducials—delivers high accuracy under uniform lighting but deteriorates when illumination is overly strong/weak or when the tag is partially occluded. The color-marker approach is most sensitive to ambient lighting and background similarity, with severe changes readily causing false positives/negatives. As target scale shrinks and pixel resolution drops with distance, these effects are amplified: YOLOv11 shows only mild error growth, ArUco remains stable provided the tag size is sufficient, and the color method exhibits the largest increase.

Beyond the above factors, we find that glare is the dominant error driver, followed by welding fume and clutter; these can be mitigated, respectively, with a polarizer plus narrow band-pass and short exposure, local fume extraction with temporal filtering, and dynamic ROI with hard-negative augmentation.

### 5.2. Application-Oriented Results and Pedagogical Implications

The engineering transferability and educational value of YLGS lie in its end-to-end chain of “vision detection → active ranging → multi-sensor fusion → closed loop control”, which mirrors the workflow of intelligent manufacturing and maps directly to process safety, in-line inspection, and quality control. In welding stations, rapid torch tip localization combined with depth feedback enables real-time correction of trajectory deviations, reducing manual intervention and rework while maintaining stable operation under challenging conditions. The hardware/software stack is modular, facilitating integration with existing PLC/HMI systems or shop-floor vision platforms and thereby lowering the barrier from lab prototype to pilot-line deployment.

For engineering education, YLGS offers a project-based, cross-disciplinary vehicle that supports completion of a full engineering cycle under realistic constraints: algorithm design, sensor calibration and error modeling, system integration, performance verification, and safety compliance. Learning outcomes can be assessed with objective metrics and complemented by evidence-based reflection using calibration logs, error budgets, versioned code, and experiment records. This promotes systems thinking, mechatronic co-design, and engineering ethics, alongside teamwork. With a reusable open-source software stack and controllable hardware costs, the framework is also well suited to capstone projects and industry–academia collaborations, helping students translate theory into verifiable engineering implementations.

## 6. Conclusions

This paper presents YLGS, a closed loop visual localization system that integrates monocular vision, laser ranging, and galvo control for precise welding torch tip positioning. By combining a YOLOv11-based detector with active ranging, the system achieves real-time millimeter-level accuracy under challenging conditions and outperforms color-based and ArUco-based baselines in both accuracy and robustness. The closed loop iteratively refines pose estimates, enabling low latency and stable tracking without physical fiducials or structured light. YLGS can also serve as a reusable resource for project-based learning and authentic assessment in engineering education. Future work will target full 6-DOF pose estimation, fusion with heterogeneous sensors, network and multi-target tracking optimization, and broader applications to diverse robotic tool-tracking tasks.

## Figures and Tables

**Table 1:** Camera errors.

Distance (cm)	Left- Measured Depth (cm)	Center- Measured Depth (cm)	Right- Measured Depth (cm)	Depth Error Left (cm)	Depth Error Center (cm)	Depth Error Right (cm)
90	89.826	90.003	90.189	0.174	0.003	0.189
100	99.831	100.192	100.172	0.169	0.192	0.172
110	110.186	109.994	109.842	0.186	0.006	0.158
120	120.214	120.307	119.743	0.214	0.307	0.257
130	130.345	130.382	130.313	0.345	0.382	0.313
140	140.513	140.458	140.481	0.513	0.458	0.481
150	150.587	150.512	150.674	0.587	0.512	0.674

**Table 2:** Ablation study of individual modules. (✔ = module enabled; blank = not used.)

Variant	CBAM	DySample	ASFFHead	Precision (%)	Recall (%)	mAP@0.5 (%)	mAP@0.5:0.95 (%)
Baseline				67.4	69.8	73.2	43.6
A	✔			68.7	70.7	74.8	45.4
B	✔	✔		69.4	71.3	75.5	44.9
C	✔	✔	✔	70.3	72.8	76.8	46.3

**Table 3:** Error statistics of the color-marker method (simulated deviation between torch tip estimate and camera ground truth, unit: mm).

Groups	Distance (m)	RME (mm)	MAE (mm)	Maximum Error (mm)
Group 1	0.9	6.27	6.87	9.34
Group 2	1.0	5.54	5.16	8.95
Group 3	1.1	7.07	6.60	10.16
Group 4	1.2	8.51	7.79	12.86
Group 5	1.3	8.45	7.93	11.58
Group 6	1.4	10.78	10.51	15.12
Group 7	1.5	13.54	12.97	17.20
Average	/	8.59	8.26	12.17

Note: This table reports the color-marker method. The error statistics indicate relatively large errors; this method serves as the baseline in this paper.

**Table 4:** Error statistics of the ArUco marker method (simulated deviation between torch tip estimate and camera ground truth, unit: mm).

Groups	Distance (m)	RMSE (mm)	MAE (mm)	Maximum Error (mm)
Group 1	0.9	4.66	4.22	7.19
Group 2	1.0	4.92	4.51	8.45
Group 3	1.1	5.40	5.07	7.38
Group 4	1.2	5.96	5.56	9.53
Group 5	1.3	6.32	6.05	9.25
Group 6	1.4	7.75	7.32	10.87
Group 7	1.5	8.64	8.24	11.12
Average	/	6.24	5.85	9.11

Note: The ArUco scheme’s error statistics indicate that its RMSE and MAE values are consistently lower than those of the color-marker scheme across all distance groups, with smoother variation as distance increases.

**Table 5:** Error statistics of Scheme III (YOLO–LaserGalvo) (simulated data; unit: mm).

Groups	Distance (m)	RMSE (mm)	MAE (mm)	Maximum Error (mm)
Group 1	0.9	3.19	2.92	5.64
Group 2	1.0	3.76	3.45	5.18
Group 3	1.1	4.01	3.87	4.51
Group 4	1.2	4.63	4.24	6.73
Group 5	1.3	5.13	4.79	5.49
Group 6	1.4	5.82	5.31	7.38
Group 7	1.5	6.79	6.46	7.02
Average	/	4.76	4.43	5.99

Note: Simulated error statistics indicate that all metrics of Scheme III are lower than those of the other two schemes over 0.9–1.5 m. The average RMSE is 4.76 mm in this interval, thus outperforming the color method (8.59 mm) and ArUco (6.24 mm).

## Data Availability

The raw data supporting the conclusions of this article will be made available by the authors upon request.
